# Differences in pain, disability, and psychological function in low back pain patients with and without anxiety

**DOI:** 10.3389/fphys.2022.906461

**Published:** 2022-11-03

**Authors:** Ying Jiang, Yizu Wang, Rui Wang, Xiaogang Zhang, Xueqiang Wang

**Affiliations:** ^1^ School of Rehabilitation Medicine, Binzhou Medical University, Yantai, Shandong, China; ^2^ Department of Rehabilitation Medicine, Ruijin Hospital, Shanghai Jiaotong University School of Medicine, Shanghai, China; ^3^ Department of Sport Rehabilitation, Shanghai University of Sport, Shanghai, China; ^4^ Department of Rehabilitation Medicine, Shanghai Shangti Orthopaedic Hospital, Shanghai, China

**Keywords:** low back pain, anxiety, disability, psychology function, correlation

## Abstract

**Objectives:** Non-specific low back pain affects people of all ages and is a leading contributor to disease burden worldwide. Chronic low back pain (LBP) reduces working hours, increases comorbidities, and increases rehabilitation needs. The aim of this study was to evaluate whether there were differences in pain, dysfunction, and psychological factors between two groups. The supplementary demonstrated the relationship between these influencing factors and anxiety.

**Methods:** A cross-sectional study was designed to analyze the differences in pain, disability, and psychological function in non-specific LBP patients with and without anxiety. In total, 60 subjects were divided into two groups based on self-rated anxiety scores: 30 patients with SAS score ≥50 were in the low back pain with anxiety group, and 30 for the LBP without anxiety group with SAS score <50. The pain intensity was assessed using the Visual Analog Scale; psychological function, using the Pain Anxiety Symptoms Scale, the Tampa Scale for Kinesiophobia, and the Fear Avoidance Beliefs Questionnaire; functional disability, using the Oswestry Disability Index and the Roland–Morris Disability Questionnaire; quality of life using 36-Item Short-Form Health Survey questionnaire; and the quality of sleep using Pittsburgh Sleep Quality Index, and the relationships between variables and anxiety scores were estimated using Spearman correlation analysis.

**Results:** A total of 60 participants were enrolled after self-rated anxiety was assessed and the full investigation was finished. The analyses showed significant differences of pain intensity (*p* = 0.034, disability (ODI, *p* = 0.007; RMDQ, *p* = 0.012) and psychological function (TSK, *p* = 0.000; PASS, *p* = 0.009; FABQ, *p* = 0.000; SF-36, *p* = 0.000; and PSQI, *p* = 0.000) between the two groups. Spearman correlation analysis showed that the anxiety score had significant positive correlations with functional disability (ODI, *p* = 0.004 and 95% CI = 0.112–0.573; RMDQ, *p* = 0.003, 95% CI = 0.135–0.586) and psychological function (TSK, *p* = 0.001, 95% CI = 0.174–0.612), excellent positive correlation with quality of sleep (PASS, *p* = 0.025, 95% CI = 0.031–0.512), and strongly negative correlations with the quality of life (SF-36, *p* = 0.000, 95% CI = 0.761–0.433).

**Conclusion:** We recognized that anxiety in low back pain patients was mainly due to interaction with the intensity of pain, disability level, and a mass of psychological function. The future research direction could be to alleviate the anxiety on the comprehensive efficacy of patients with low back pain.

## Introduction

In recent years, low back pain (LBP) has become a common cause of musculoskeletal disorders which leads to disability, reduced work hours, and the need for rehabilitation (Chenot et al., 2017). In 2015, LBP was responsible for 60.1 million person-years lived with disability (YLD) (GBD 2015 Disease et al., 2016). The Global Burden of Diseases (GBDs) updated the prevalence data of the global burden of more than 300 diseases in 2019; as the proportion of LBP reached 39.77%, it ranked first among the risk factors of YLD in the world (GBD 2019 Diseases and Injuries Collaborators, 2020). It places a serious financial burden of healthcare both in the family and society (Urits et al., 2019). There are few articles that have estimated the global cost of LBP, and some changes in policy may even affect the cost of LBP; however, there is no denying that the economic impact of LBP is considerable, especially in high-income countries (Hartvigsen et al., 2018). LBP not only increases with age, but it is also becoming more common in adolescents (Patrick et al., 2014). That is why LBP has been steadily given great attention. Low back pain can be divided into specific low back pain and non-specific low back pain according to the identified etiology. Non-specific low back pain is the most common form of low back pain (Chenot et al., 2017). Although LBP patients generally recover in a short period of time, some people develop chronic low to moderate pain, intermittent or persistent (Vlaeyen et al., 2018). However, recurrence is also common, and LBP can become persistent and disabling (Hartvigsen et al., 2018). In addition to disability, psychology-related problems, such as anxiety, depression, and sleep disturbance, also trouble the patients of LBP (Alleva et al., 2016).

Any dysfunction has a variety of psychological effects, and many studies have shown that people with LBP have somatic, emotional, and psychological problems (Maniadakis and Gray, 2000; Pincus et al., 2002; Edmond et al., 2010; Adilay et al., 2018; Singhal et al., 2021); these factors influence the effectiveness of treatment outcomes (Pinheiro et al., 2016; Bijker et al., 2020). Psychological disturbances may cause chronic LBP, or result from it (Kjellgren et al., 2007). These disturbances include anxiety, depression, anger, and fear (Tekur et al., 2012). Chronic LBP is strongly correlated with anxiety (Kim et al., 2006). Patients with LBP have a higher likelihood of experiencing anxiety (9.5% versus 6.2%), followed by somatization (14.9% versus 8.3%) and depression (13.7% versus 8.5%) (Bener et al., 2013). Anxiety is associated with increased use of healthcare resources (Bailes et al., 2021). As studied previously, the majority of LBP patients who did not recover from physical therapy interventions were strongly associated with anxiety, depression, and fear of movement (George and Beneciuk, 2015). This evidence suggests that low back pain and anxiety are bidirectional and interactive. Therefore, more and more researchers are paying attention to LBP patients with anxiety, trying to improve the comprehensive symptoms of LBP by reducing anxiety. Pain relief and function improvement are connected with a reduction in anxious states (Joyce et al., 2021). Sleep disorders, pain-related dysfunction, and anxiety in chronic LBP patients can be improved by stabilization exercises (Akodu and Akindutire, 2018). Nevertheless, there are few articles about the influencing factors of anxiety in patients with LBP.

Awareness of the relationship between anxiety and characteristics such as pain, dysfunction, and mental factors in LBP patients provides important information that could be used to predict treatment effects and prognosis, and also help select the optimal treatment method and self-management. The Lancet Low Back Pain Series working group calls for the concept of living well with low back pain (Buchbinder et al., 2018): this means understanding and managing one’s health is important for maintaining the function of LBP patients. However, few studies focus on the differences in pain, function, and other psychological factors between low back pain patients with and without anxiety. Thus, there is a gap in the comprehensive treatment of patients with low back pain. Many studies have found the problem of anxiety in patients suffering from low back pain for a long time, but there is no study that discusses the differences in many functions of low back pain patients with or without anxiety. So, the objective of this study was to evaluate whether there were differences in pain, dysfunction, and psychological factors between the two groups. We hypothesized that low back pain patients with or without anxiety would differ in many functions. This study also supplementarily demonstrated the relationship between these influencing factors and anxiety.

## Materials and methods

### Study design

This was a cross-sectional study to analyze differences in pain, disability, and psychological function in LBP patients with and without anxiety. The investigation was performed in the Rehabilitation of Shanghai Shangti Orthopedic Hospital, Shanghai, China, from June 2021 to January 2022. The data collection was completed by the author RW, reducing the potential for error arising from different operators. All participants understood the process of the study and signed informed consent prior to the assessment. The Ethics Committee of Shanghai University of Sport approved this study (number 2018069).

### Participants

The calculation of sample size was obtained from previous studies that reported the pain difference of low back pain patients with anxiety, through G-power (one tail; effect size, 0.65; ɑ, 0.05; power, 0.80; N2/N1, 1) (Stubbs et al., 2016). The results were 30 for each group, and the actual power was 0.800; finally, 60 participants were needed to complete the study. The Self-rating Anxiety Scale (SAS) was used to classify the participants into two groups: 30 for LBP with anxiety, and 30 for LBP without anxiety. The inclusion criteria are as follows: 1) all the participants were Chinese adults aged 18–65 years who suffered from non-specific chronic LBP (Chou et al., 2007), 2) the persistent presence of LBP lasting at least 50% of the time during the past 6 months, and 3) good cognition and ability to cooperate to complete various questionnaires. The patients with the following conditions were excluded: 1) specific LBP, such as spinal stenosis, severe scoliosis, and ankylosing spondylitis; 2) lumbar disability and/or pain caused by other conditions; 3) other severe and/or unstable chronic diseases; and 4) pregnant or lactating.

## Measurements

### Pain intensity

The pain intensity was assessed with a Visual Analog Scale (VAS), which is a self-reported scale using a 10-cm line to verbally describe the pain status indicated at each end (Dworkin et al., 2005). The higher the score, the more severe the pain (Chiarotto et al., 2019). VAS is popularly used in the assessment of low back pain because of its good reliability and validity (Shafshak and Elnemr, 2021). The study required participants to record their resting and severe VAS score according to their pain experience.

## Psychological function

### Zung Self-rating Anxiety Scale

The Zung Self-rating Anxiety Scale (SAS) is a commonly used self-rating scale for mental problems, which consists of 20 items that include both psychological and somatic anxiety symptoms (Dunstan and Scott, 2020). Each item is rated 1–4, where 1 means none, or a little of the time, and 4 means most or all of the time. The total score of SAS ranged from 20–80; a higher score indicates severe anxiety (Jegede, 1977; Dunstan et al., 2017). The standard score is a rough score (sum of each item) multiplied by 1.25, and anxiety is defined using the cut-off score of 50; the scale has good reliability (Cronbach *α* = 0.92) and can be used as an effective assessment tool for an anxiety state (Zung, 1971).

### The Pain Anxiety Symptoms Scale

The Pain Anxiety Symptoms Scale (PASS) was used to measure the fear of pain and pain-related sensations (McCracken et al., 1992). The 40-item Pain Anxiety Symptoms Scale (PASS-40) comprises four subscales: cognitive anxiety (CA), fearful appraisal (FA), escape/avoidance (EA), and physiological anxiety (PA). Each item is scored from 0 (never) to 5 (always) (Babel, 2017). The higher the score, the greater the fear of pain and severe anxiety. PASS obtained good reliability (Cronbach *α* = 0.92, ICC = 0.90) in a simplified Chinese version (Zhou et al., 2017).

### The Tampa Scale for Kinesiophobia

The Tampa Scale for Kinesiophobia (TSK) was developed to assess fear of movement-related pain in patients with musculoskeletal pain. TSK-17 is widely used; it contains 17 items, each item rated from 1 (strongly disagree) to 4 (strongly agree); and total scores vary from 17 to 68. A higher score indicates more fear of movement (Rosenbloom et al., 2020). The TSK has demonstrated good test–retest reliability (Swinkels-Meewisse et al., 2003) and good internal consistency (Cronbach alpha = 0.68–0.86) (Vlaeyen et al., 1995; Swinkels-Meewisse et al., 2003).

### The Fear-Avoidance Beliefs Questionnaire

The Fear-Avoidance Beliefs Questionnaire (FABQ) focused on patients who thought about how physical activity (FABQ-P) and work (FABQ-W) affected their current low back pain (Waddell et al., 1993). FABQ is a 16-item self-reported questionnaire with each item rated from 0 to 6, the total score is 96, and higher values reflect increased levels of fear-avoidance beliefs (Wertli et al., 2014). It has been proved to have excellent internal consistency: for the FABQ total, *α* = 0.82; FABQ-P, *α* = 0.75; and FABQ-W, *α* = 0.85 (Pei et al., 2010). Understanding pain-related fears from a common sense perspective may allow physical therapists to provide a path to recovery for LBP patients and reduce fear by changing their understanding of pain (Bunzli et al., 2017).

## Disability

### The Oswestry Disability Index

The Oswestry Disability Index (ODI) is a self-assessment measurement scale primarily designed to assess the degree of disability in patients with low back pain (Poder and Carrier, 2021). Ten evaluation items are included in the ODI scale: personal care, pain intensity, walking, lifting, standing, sitting, sleep, work, traveling, and social life. Each item is rated from 0 (no pain at all) to 5 (extreme pain) on six levels (van Hooff et al., 2015; Kim et al., 2016). A high total score indicates poor function. Different versions of ODI show excellent test–retest reliability (ICC = 0.70–0.97) (Yu et al., 2016a; Gamus et al., 2016) and good internal consistency (0.72–0.97) (Aiyegbusi et al., 2017; Sandal et al., 2021).

### The Roland–Morris Disability Questionnaire

The Roland–Morris Disability Questionnaire (RMDQ) was designed to evaluate dysfunction in patients with LBP, which comprises 24 questions closely related to LBP selected from 136 questions in the Sickness Impact Profile (Roland and Morris, 1983). The RMDQ can assess dysfunction in patients with LBP within 24 h, including walking, standing, bending, staying in bed, sleeping, dressing, daily activities, and self-care. The question is answerable by “yes” for 1 point and “no” for 0 point, and the total score for all questions is the actual score from the lowest 0 to the highest 24. A higher score shows a higher level of dysfunction (Beaton et al., 2000). RMDQ has excellent reliability (ICC = 0.91), good internal consistency (Cronbach’s alpha = 0.88) (Chala et al., 2021), and a moderate-to-excellent validity (Chiarotto et al., 2016).

### Quality of life

The 36-Item Short Form Health Survey questionnaire (SF-36) is a very common scale for evaluating health-related quality of life (HRQoL) (Lins and Carvalho, 2016). The scale consists of 36 items that cover eight subscales related to physical and social functions, including pain and mental conditions (Ware and Sherbourne, 1992). The score of each subscale ranges from 0 to 100, and higher scores revealed better HRQoL (Bunevicius, 2017). It has been proved to have good cultural adaptation and validation (Li et al., 2003).

### Quality of sleep

The Pittsburgh Sleep Quality Index (PSQI) is a self-reported questionnaire to assess sleep quality and disturbances for more than 1 month. The questionnaire has seven subscale scores of nineteen individual items (Buysse et al., 1989). Each subscale is scored from 0 to 3. The total PSQI score may range from 0 to 21, with higher scores showing poorer sleep quality (Telford et al., 2019). The reliability and validity of different versions were proved (Mollayeva et al., 2016).

### Statistical analysis

Statistical analysis was conducted using SPSS version 23.0 (IBM Corporation, Armonk, NY, United States). The graph was drawn with GraphPad. We performed descriptive statistics on the baseline characteristics of two groups, after normal distribution was checked (*p* > 0.05); all numerical variables of the normal distribution are represented as mean ± standard deviation (SD), while abnormal distribution, in terms of the interquartile range (Q1–Q3). The chi-square test was used for the comparison between groups when the variables were classified variables. *T*-test was used to compare the differences in SF-36, PASS, FABQ-total, and FABQ-W between patients with and without anxiety because of its normal distribution. A non-parametric test was used to compare the differences in VAS, ODI, RMDQ, TSK, and PSQI between patients with and without anxiety, due to abnormal distribution. Spearman correlation analysis was carried out to analyze the relationships between pain intensity, disability, psychological function, quality of life and quality of sleep, and anxiety. The Spearman correlation coefficient might range from +1 (positive correlation) to −1 (negative correlation), as 0 indicates no association between the two variables, and greater values indicate stronger associations between variables; values were interpreted as little relationship, fair, moderate, and excellent relationship with values <0.25, 0.25–0.50, 0.50–0.75, and ≥0.75, respectively (Emami et al., 2018).

## Result

A total of 60 participants were enrolled after SAS assessment and the full investigation was finished; 30 participants’ SAS score were lower than 50, and the other 30 participants’ SAS score were higher than 50. Table 1 shows the demographics and characteristics of all patients with chronic LBP. There were no differences in the baseline information of the two groups.

**TABLE 1:** Demographics and characteristics of patients with low back pain.

General characteristic	Anxiety group (*n* = 30)	Non-anxiety group (*n* = 30)	Z/χ^2^	*p*-value
Gender (male)	10 (33.3%)	17 (56.7%)	3.300	0.069
Age∼(year)	26.56 (24.0–48.8)	27.00 (23.75–36.00)	−0.636	0.525
Height^(cm)	167.05 ± 7.32	167.61 ± 8.84	−0.444	0.657
Weight^	64.52 ± 12.02	67.68 ± 12.95	−0.917	0.359
BMI^	23.04 ± 3.69	23.87 ± 3.09	−1.065	0.287
Moderate intensity physical activity per week				
＜150 min	11 (36.7%)	8 (26.7%)	0.764	0.683
150–300 min	7 (23.3%)	9 (30.0%)		
＞300 min	12 (40.0%)	13 (43.3%)		
Duration of LBP, years				
3–5 years	15 (50.0%)	18 (60.0%)	2.606	0.626
5–10 years	8 (26.7%)	8 (26.7%)		
10–15 years	3 (10.0%)	3 (10.0%)		
15–20 years	2 (6.7%)	1 (3.3%)		
>20 years	2 (6.7%)	0 (0%)		
Nature of pain				
Soreness and swelling	20 (66.7%)	21 (70.0%)	2.624	0.269
Radiated	3 (10.0%)	6 (20.0%)		
Pins and needles	7 (23.3%)	3 (10.0%)		
Aggravated behaviors				
Sitting	4 (13.3%)	6 (20.0%)	8.222	0.412
Standing	3 (10.0%)	7 (23.3%)		
Walking	10 (33.3%)	8 (26.7%)		
Bending	3 (10.0%)	0 (0%)		
Squatting	4 (13.3%)	2 (6.7%)		
Upstairs	1 (3.3%)	2 (6.7%)		
Downstairs	3 (10.0%)	1 (3.3%)		
Postural change	1 (3.3%)	1 (3.3%)		
Others	1 (3.3%)	3 (10.0%)		
24 h pattern				
Getting worse	13 (43.3%)	6 (20.0%)	4.888	0.180
Alleviating	5 (16.7%)	11 (36.7%)		
No change	8 (26.7%)	9 (30.0%)		
Others	4 (13.3%)	4 (13.3%)		
Frequency of pain	9 (3.75–15)	9 (2–20)	−0.446	0.655
Average duration of a day, hour	5 (2.75–8.00)	4.00 (1.00–6.25)	−1.281	0.200
Total leisure hours a day	8.00 (7.00–10.00)	7.50 (6.00–9.00)	−1.324	0.186
Medication situation				
No	17 (56.7%)	25 (83.3%)	7.643	0.054
Drugs for pain	7 (23.3%)	1 (3.3%)		
Adjuvant therapy	5 (16.7%)	2 (6.7%)		
Others	1 (3.3%)	2 (6.7)		

BMI, body mass index; RMB, renminbi; ∼, non-normally distributed data are presented as median (Q1–Q3); ^, normally distributed data are presented as number of participants (%). *p*-values come from the Mann–Whitney *U*-test for quantitative data and the chi-square test for qualitative data.

**FIGURE 1:**
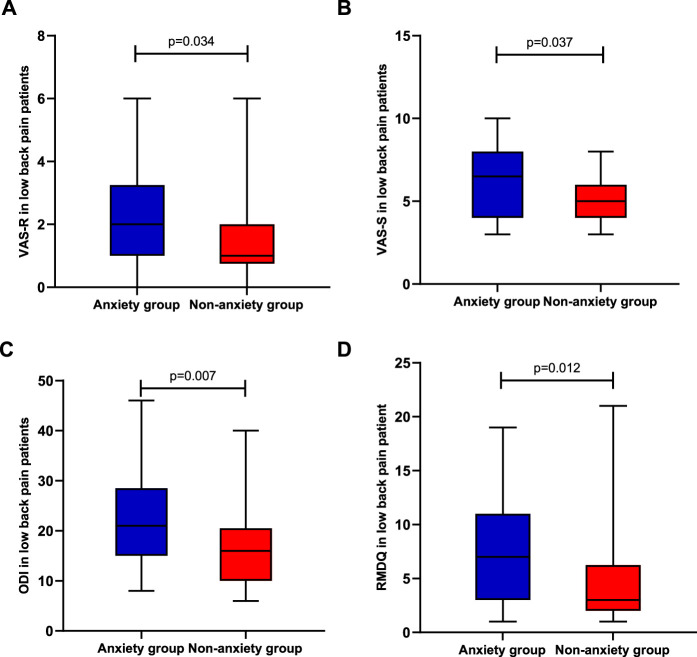
Differences in pain and disability between the anxiety group (*n* = 30) and non-anxiety group (*n* = 30); **(A)** pain intensity in rest; **(B)** pain intensity in severe pain; **(C)** Oswestry Disability Index; and **(D)** Roland–Morris Disability Questionnaire.

**FIGURE 2:**
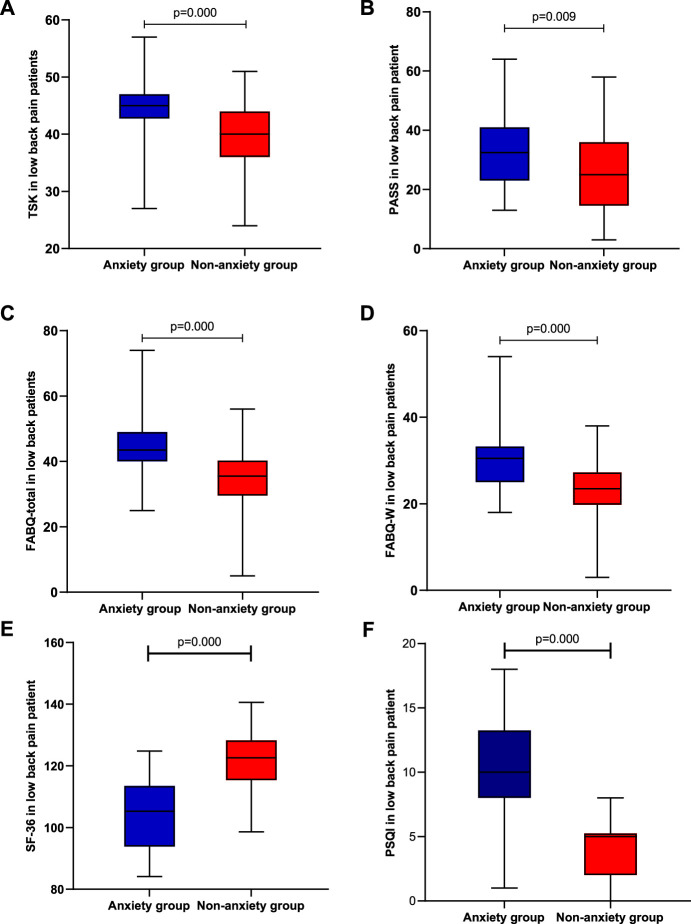
Differences in psychological function, quality of life, and quality of sleep between the anxiety group (*n* = 20) and non-anxiety group (*n* = 20); **(A)** Tampa Scale for Kinesiophobia; **(B)** Pain Anxiety Symptoms Scale; **(C)** Fear-Avoidance Beliefs Questionnaire’s total score; **(D)** Fear-Avoidance Beliefs Questionnaire in this work; **(E)** 36-Item Short Form Health Survey questionnaire; and **(F)** Pittsburgh Sleep Quality Index.

To examine whether two groups (low back pain with anxiety and low back pain without anxiety) differ in pain intensity, disability, and psychological function, we performed *t*-tests on two separate samples. Means, standard deviation, or interquartile range are presented in Figures 1, 2. The analyses show significant differences in pain intensity, disability, and psychological function between two groups with *p*-value less than 0.01 in SF-36 (z = 2.122, *p* = 0.000), PASS (z = 0.042, *p* = 0.009), FABQ total (z = 0.104, *p* = 0.000), FABQ-W (z = 0.109, *p* = 0.000), ODI (f = −2.686, *p* = 0.007), PSQI (f = −5.980, *p* = 0.000), and TSK (f = −3.773, *p* = 0.000), and less than 0.05 in RMDQ (f = −2.513, *p* = 0.012), FABQ-PA (f = −1.967, *p* = 0.049), VAS-R (f = 2.125, *p* = 0.034), and VAS-S (f = −2.084, *p* = 0.037). It indicates that LBP patients with anxiety had higher pain intensity and disability level, seemed more afraid of pain and pain-related activity, and had lower quality of life and sleep.

The Spearman correlation analysis was conducted to reveal the relationships between anxiety and pain intensity, disability, and psychological function in all patients. The results in Table 2 show that SAS score had significant positive correlations with PASS (*r* = 0.290, *p* = 0.025), ODI (*r* = 0.366, *p* = 0.004), RMDQ (*r* = 0.382, *p* = 0.003), PSQI (*r* = 0.738, *p* = 0.000), TSK (*r* = 0.416, *p* = 0.001), FABQ-W (*r* = 0.402, *p* = 0.001), FABQ total (*r* = 0.375, *p* = 0.003), and VAS-S (*r* = 0.413, *p* = 0.001), and strongly negative correlations with SF-36 (*r* = 0.624, *p* = 0.000). The quality of life and sleep were significantly correlated with anxiety symptoms of LBP.

**TABLE 2:** Spearman correlation analysis between SAS and each variable.

		ODI	RMDQ	SF-36	PSQI	PASS	TSK	FABQ-total	FABQ-W	FABQ-P	VAS-S	VAS-R
SAS	Spearman’s rho	0.367	0.383	−0.624	0.734	0.290	0.416	0.377	0.404	0.198	0.249	0.412
	*p*-value	0.004	0.003	0.000	0.000	0.025	0.001	0.003	0.001	0.129	0.055	0.001
	95% CI	0.112–0.573	0.135–0.586	−0.761–0.433	0.585–0.835	0.031–0.512	0.174–0.612	0.129–0.581	0.160–0.602	−0.067–0.437	−0.013–0.480	0.169–0.608

SAS, self-rated anxiety; ODI, the Oswestry Disability Index; RMDQ, the Roland–Morris Disability Questionnaire; SF-36, The 36-Item Short Form Health Survey questionnaire; PSQI, the Pittsburgh Sleep Quality Index; PASS, the Pain Anxiety Symptoms Scale; TSK, the Tampa Scale for Kinesiophobia; FABQ, Fear-Avoidance Beliefs Questionnaire; FABQ-P, Fear-Avoidance Beliefs Questionnaire on physical activity; FABQ-W, Fear-Avoidance Beliefs Questionnaire on Work; VAS-S, Visual Analog Scale of severe pain; VAS-R , visual analog scale at rest.

## Discussion

Patients with low back pain who suffer from pain and dysfunction for a long time will be affected both mentally and physically. In recent years, it has become important to explore the related influencing factors of low back pain patients from multiple dimensions under the biopsychosocial model. This cross-section investigated the relationship between pain, disability, psychological function, and anxiety in LBP patients. We found that there were statistically significant differences between LBP patients with anxiety and those without anxiety in these aspects. However, severe pain score was not correlated with anxiety, and daily life quality and sleep quality were strongly correlated with anxiety, while TSK and FABQ-work were moderately correlated, and other factors were weakly correlated with anxiety.

Pain intensity is a common outcome measurement to assess the effect of therapy and quantify the degree of chronic LBP. It is a personal experience influenced by multidimensional factors, such as genetic, psychological, cultural, and social factors (Williams and Craig, 2016). There is a positive correlation between pain and anxiety in LBP patients, and the results of this study are in keeping with previous studies (Pakarinen et al., 2014; Stubbs et al., 2016), the possible mechanism is as follows: 1) multiple regions of the brain evoke pain responses through pathologic pathways of peripheral and central sensory input (e.g., sensory, anxiety, and other emotional perception), the descending pathways can promote or inhibit the nociceptive information because the descending pathway is a pain-regulating pathways lie because of this mechanism, that pain is affected by top–down vary and dynamic factors (such as feel the danger, anxiety, emotion, pain memory) that determine the result of pain experiences (Voscopoulos and Lema, 2010). 2) In chronic pain, this activity shifts to specific networks in the brain that process emotions (e.g., prefrontal cortex) (Hashmi et al., 2013); because of the interaction between physical and psychological systems, these changes affect how pain is expressed and experienced (Kucyi and Davis, 2015). However, the results of this study show that the most severe pain score is not correlated with anxiety (1B); we believe this happened possibly because anxiety is the result of excessive internal conflict that threatens oneself when experiencing low self-esteem and frustration for a long time, while patients tend to focus on the pain itself when experiencing severe pain.

ODI and RMDQ are popular in assessing dysfunction in patients with LBP; in recent years, many studies have focused on the relationship between psychological factors and disability. They found that dysfunction was weakly associated with mood (e.g., anxiety and depression) (Reneman et al., 2008; La Touche et al., 2019), which is consistent with the results of this study. If there is no improvement in functional activities or mobility over a long period of time, it can greatly increase the patient’s chances of anxiety; in contrast, low back pain patients accompanied by anxiety are more likely to relapse after relief of pain and dysfunction because anxiety is a risk factor for recurrence of low back pain. Compared to LBP without anxiety, those with anxiety focused more on negative thoughts and opinions about themselves, while the former focused on their health issues. For clinical workers, the treatment concepts and methods of the two groups should be different, and there are no articles about the treatment of the two groups at present.

Our results show that TSK and FABQ-W are moderately correlated with anxiety in LBP patients (2A and 2D). Kinesiophobia is a form of exercise protection; its value is that it can predict an individual’s degree of disability by comparing it to the intensity and duration of clinical symptoms, pain, and anxiety (Trocoli and Botelho, 2016). The main reasons for psychological factors affecting patients’ kinesiophobia are as follows: negative emotions, such as anxiety and depression, can exacerbate the patient’s fear of pain and exercise, which in turn can cause the patient to reduce rehabilitation exercise and daily activities; decreased activity reduces muscle strength and aerobic capacity, exacerbating negative emotions like irritability and frustration. Therefore, negative emotions and fear of movement are format interaction effects which result in a vicious circle. In addition, the work-related fears in LBP patients with anxiety are more evident: the possible reason may be that the work environment or occupational activities would cause low back pain symptoms; moreover, more attention should be paid to reducing the associated costs due to the high proportion of patients experiencing anxiety (Buchbinder and Jolley, 2004); furthermore, the sample population of our study is young and relatively tolerant against higher work-related stress.

Quality of life is a very important outcome of LBP patients, which is affected by multiple dimensions, primarily mobility, followed by anxiety or depression, and the conclusions of this study complement the findings by Cedraschi et al. (2016). The patients who have chronic musculoskeletal pain combined with psychosomatic factors (anxiety and depression) were correlated with more severe pain and greater daily activities than those who experienced pain alone. Previous studies (Bingefors and Isacson, 2004; Macfarlane et al., 2012; Hazeldine-Baker et al., 2018) have shown that chronic LBP patients with psychological disorders (mood and/or anxiety disorders) are highly associated with more severe pain and disability and dysfunctional management. In addition to the medical aspects of the disease, the quality of life is seriously impaired due to psychosocial problems, and QoL was negatively correlated with anxiety (Yeni et al., 2018).

Insomnia is very common among people with chronic pain, and is often thought to be a consequence of chronic pain, but the findings demonstrate a bidirectional relationship with pain (Eccleston et al., 2009; Pigeon et al., 2012). There is a close mutual effect between sleep disorders and central sensitization in people with chronic pain (Nijs et al., 2018). It has been proved that night insomnia can induce general hyperalgesia and anxiety state in healthy people (Schuh-Hofer et al., 2013). In this study, there is a strong positive correlation between sleep disturbance and anxiety in patients with LBP (2F): higher levels of sleep disturbance corresponded to greater anxiety symptoms. The result is in line with other similar articles (Yu et al., 2016b). Disrupted circadian rhythms may be responsible for the increased risk of mood disorders. It is important to assess sleep disturbances in LBP patients due to robust associations with fatigue, depression, and anxiety.

## Limitations

Despite the new findings in this study, the following limitations still exist: 1) as a cross-sectional study, this study cannot determine causality; 2) a small sample size was included, and the conclusion about the predictive ability of the included factors was limited. Therefore, a longitudinal study with a larger sample size is needed to further explore the relationship and mechanism of various influencing factors in LBP patients.

### Clinical implications and future research

Although the relationship between symptoms of LBP and anxiety is very weak in addition to the quality of life and quality of sleep, the problem still commonly exists in patients with LBP; in view of this, psychotherapy should be an integral part of a multidisciplinary rehabilitation training strategies, psychological consultant for patients with anxiety, and other negative emotions control strategy of education to improve their psychological state. In terms of the treatment of chronic LBP, we should pay attention to the emotional changes of patients as soon as possible, adopt the method of multidisciplinary cooperation and management to put forward targeted psychological intervention measures, so as to reduce the pain degree of patients and improve the psychological status of patients.

## Conclusion

In this investigation, it was possible to identify some of the psychological and physiological characteristics involved in low back pain anxiety. To sum up, we recognized that anxiety in LBP patients interacts with the intensity of pain, disability level, and a mass of psychological function; therefore, these factors influence the effectiveness of treatment, and clinicians are recommended to pay attention to and offer the comprehensive evaluation and individual intervention.

## Data Availability

The raw data supporting the conclusion of this article will be made available by the authors, without undue reservation.
